# Tandem Mass Tagging (TMT) Reveals Tissue-Specific Proteome of L4 Larvae of *Anisakis simplex* s. s.: Enzymes of Energy and/or Carbohydrate Metabolism as Potential Drug Targets in Anisakiasis

**DOI:** 10.3390/ijms23084336

**Published:** 2022-04-14

**Authors:** Robert Stryiński, Jesús Mateos, Mónica Carrera, Jan Paweł Jastrzębski, Iwona Bogacka, Elżbieta Łopieńska-Biernat

**Affiliations:** 1 Department of Biochemistry, Faculty of Biology and Biotechnology, University of Warmia and Mazury in Olsztyn, 10-719 Olsztyn, Poland; 2 Clinical Pharmacology Group, Health Research Institute of Santiago de Compostela (IDIS), Santiago de Compostela, 15-706 A Coruña, Spain; jesus.mateos.martin@sergas.es; 3 Department of Food Technology, Marine Research Institute (IIM), Spanish National Research Council (CSIC), 36-208 Vigo, Spain; 4 Department of Plant Physiology, Genetics and Biotechnology, Faculty of Biology and Biotechnology, University of Warmia and Mazury in Olsztyn, 10-719 Olsztyn, Poland; bioinformatyka@gmail.com; 5 Department of Animal Anatomy and Physiology, Faculty of Biology and Biotechnology, University of Warmia and Mazury in Olsztyn, 10-719 Olsztyn, Poland; iwonab@uwm.edu.pl

**Keywords:** *Anisakis simplex*, L4 stage larvae, intestine, cuticle, folliculin, oxoglutarate dehydrogenase, proteomics, LC-MS/MS

## Abstract

*Anisakis simplex* s. s. is a parasitic nematode of marine mammals and causative agent of anisakiasis in humans. The cuticle and intestine of the larvae are the tissues most responsible for direct and indirect contact, respectively, of the parasite with the host. At the L4 larval stage, tissues, such as the cuticle and intestine, are fully developed and functional, in contrast to the L3 stage. As such, this work provides for the first time the tissue-specific proteome of *A. simplex* s. s. larvae in the L4 stage. Statistical analysis (FC ≥ 2; *p*-value ≤ 0.01) showed that 107 proteins were differentially regulated (DRPs) between the cuticle and the rest of the larval body. In the comparison between the intestine and the rest of the larval body at the L4 stage, 123 proteins were identified as DRPs. Comparison of the individual tissues examined revealed a total of 272 DRPs, with 133 proteins more abundant in the cuticle and 139 proteins more abundant in the intestine. Detailed functional analysis of the identified proteins was performed using bioinformatics tools. Glycolysis and the tricarboxylic acid cycle were the most enriched metabolic pathways by cuticular and intestinal proteins, respectively, in the L4 stage of *A. simplex* s. s. The presence of two proteins, folliculin (FLCN) and oxoglutarate dehydrogenase (OGDH), was confirmed by Western blot, and their tertiary structure was predicted and compared with other species. In addition, host–pathogen interactions were identified, and potential new allergens were predicted. The result of this manuscript shows the largest number of protein identifications to our knowledge using proteomics tools for different tissues of L4 larvae of *A. simplex* s. s. The identified tissue-specific proteins could serve as targets for new drugs against anisakiasis.

## 1. Introduction

*Anisakis simplex* s. s. is one of the most important emerging parasitic nematodes in Europe, according to the ranking for risk management of foodborne parasites prepared for recommendations of the Food and Agriculture Organization of the United Nations (FAO) and the World Health Organization (WHO) [1,2,3]. The disease caused by the *Anisakis* genus is called anisakiasis [4,5,6] and is dangerous, well-known, common, and frequently described. It is estimated that the incidence is 0.32/100,000 worldwide [7]. The global introduction of a different cuisine, the development of better diagnostic tools, and a greater knowledge of *Anisakis* and its infection have led to significantly more anisakiasis being reported [5]. Prior to 2010, over 20,000 cases of anisakiasis were reported worldwide, with the highest prevalence (over 90%) in Japan [8,9], where 2000–3000 cases of the disease are reported annually [10]. The European countries in which cases of anisakiasis have been reported include Spain [11], Italy [12,13], France, Germany [4], Croatia [14], and Poland [15]. In addition, a recently published meta-regression analysis revealed a significant 283-fold increase in infection level of *Anisakis* spp. (average number of worms/fish) over a 53-year period from 1962 to 2015 [16]. This increase in infection level of *Anisakis* spp. may have implications for human health, marine mammal health, and fisheries profitability.

The life cycle of *Anisakis* nematodes follows the general pattern of the life cycle of nematodes, including the four larval stages L1–L4: in eggs (L1–L2) and then in intermediate or paratenic hosts (L3), and as preadults (L4) and adults in cetacean final hosts [17]. Nematode eggs are excreted in feces and embryonate in seawater. During ingestion by the first crustacean intermediate host, L2 larvae are most likely freed from the cuticle of the second stage by the action of the mouthparts and mature into L3. This allows the third stage (L3) larvae to enter the gut before settling in the hemocoel [18]. Larger invertebrates (especially copepods, euphausiids) and smaller fish are considered important second intermediate hosts, and various predatory fish species and cephalopods serve as paratenic hosts (crustaceans infected with the L3 are eaten). Whales and dolphins ingest the nematodes by eating the intermediate hosts. This complex life cycle involves a variety of hosts that are transmitted through the marine food chain [17,19]. Humans become accidentally infected by eating raw or inadequately processed L3-contaminated fish or squid [4,17,20]. Once in the human gastrointestinal tract, L3 of *A. simplex* can develop into L4, but only in exceptional cases is the immature adult stage reached [21,22,23,24,25].

As is common among parasitic nematodes with complex life cycles, the morphology of *A. simplex* varies with different stages and infected hosts. In general, these differences result from changes in diet and mode of feeding. In L3 larvae of *A. simplex*, the intestinal lumen is shrunken, and data from the literature suggest that the cuticle, a complex outer matrix covering the body of the nematode, is involved in the uptake of energy compounds (saccharides) [26,27]. After the third molt, in L4 larvae, the intestine becomes clear and begins to function [28]. Probably, after its purification, it takes over the nutritional functions from the cuticle [27]. These phenomena are very interesting and require further directional research. Moreover, from the parasitological point of view, the cuticle and the intestine are the tissues most responsible for the contact of the parasite with the host.

The cuticle and intestine are composed of and release proteins, including enzymes, that play a critical role in the introduction and development of parasitic nematodes in their hosts. To date, little is known about the proteomic composition of these specific tissues of *A. simplex*, particularly larval stage L4, which is less studied by the scientific community. Given recent developments in proteomic methods used to identify and quantify the protein composition of cells, cell secretomes, and tissue sections and whole organisms [3,29], it is not surprising that proteomic applications have gained recognition in a rapidly evolving scientific discipline such as parasitology.

Therefore, in this work, we present for the first time quantitative proteomic profiling of the specific tissues of *A. simplex* s. s. L4 larvae: cuticle (CUT) and intestine (INT), compared to the rest of the body (REST; the remains of the larval body after isolation of the cuticle and intestine), using the shotgun proteomics method based on isobaric mass labeling (TMT) with a combination of nano-high performance liquid chromatography (nLC) coupled to an LTQ-Orbitrap Elite mass spectrometer.

## 2. Results

As a result of LC-MS/MS analysis, we identified a total of 3435 proteins. These data were further processed according to selected criteria: (a) proteins classified as master proteins (2902 proteins remaining), and (b) proteins with at least 2 unique peptides (1831 proteins remaining). We used a total of 1831 proteins for further analysis (File S1). There were 1277 characterized proteins in the obtained cohort. This is the largest repository of proteins identified to our knowledge for specific tissues of L4 larvae of *A. simplex* s. s.

### 2.1. Overview of Differentially Regulated Proteins

**Figure 1:**
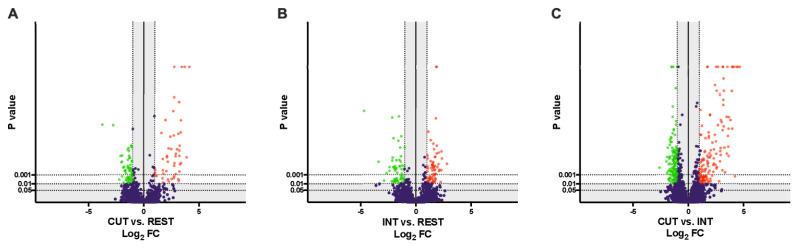
Volcano-plot representations of tissue-specific proteome of the L4 developmental stage of *Anisakis simplex* s. s. The following comparisons between the groups were visualized: (**A**) cuticle vs. the rest of the body of the L4 stage larvae, (**B**) intestine vs. the rest of the body of the L4 stage larvae, and (**C**) cuticle vs. intestine (FC ≥ 2, *p*-value ≤ 0.01). The detailed list of DRPs in each of the compared groups is presented in File S1.

After relative quantification, several filters were next applied to obtain the final list of differentially regulated proteins (DRPs): (a) at least a 2-fold change (FC ≥ 2) in normalized ratios (CUT vs. REST, INT vs. REST, CUT vs. INT), (b) ANOVA on ranks and Tukey HSD post-hoc test (*p*-value ≤ 0.01) (File S1). The volcano plot representations of DRPs are shown in Figure 1A–C.

Statistical analysis (FC ≥ 2; *p*-value ≤ 0.01) showed that 107 proteins were differentially regulated between the cuticle and the rest of the larval body at the L4 stage, of which 49 were more abundant in the cuticle and 58 in the rest of the nematode body (Figure 1A, File S1). Comparing the intestine and the rest of the body of L4 stage larvae, 123 proteins were identified as DRPs, with 67 proteins being more abundant in the intestine and 56 in the rest of the body of *A. simplex* s. s. (Figure 1B, File S1). Comparison of the individual tissues examined revealed a total of 272 DRPs, with 133 proteins more abundant in the cuticle and 139 proteins more abundant in the intestine (Figure 1C, File S1).

**Figure 2:**
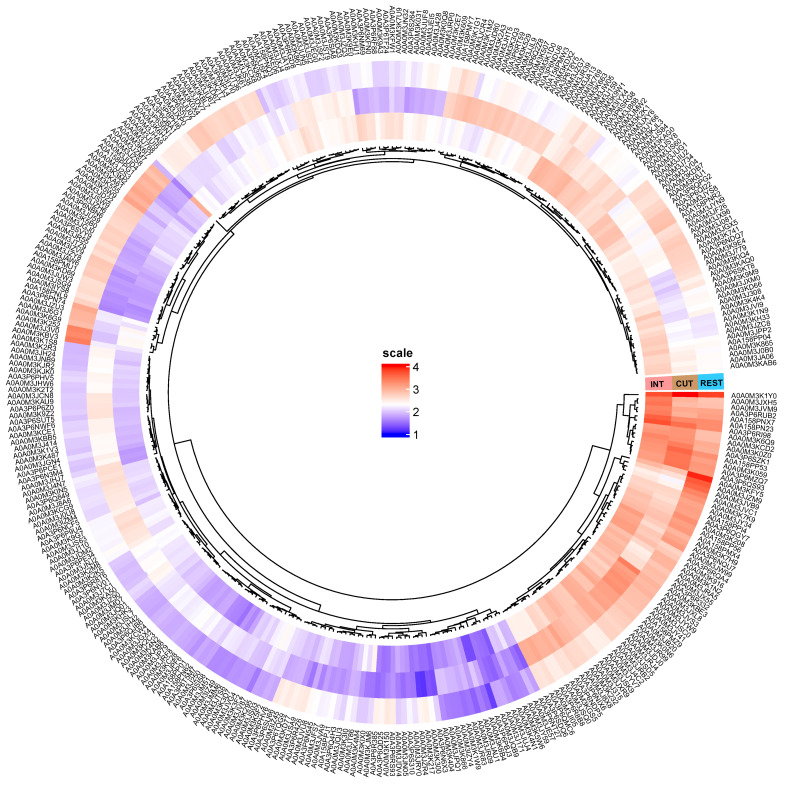
Visualization of the differentially regulated proteins (FC ≥ 2, *p*-value ≤ 0.01) between 3 tested groups: cuticle (CUT), intestine (INT), and the rest of the body of *A. simplex* s. s. (REST). Red and blue colors describe an increased and decreased abundance in the compared groups, respectively. The detailed list of DRPs in each of the compared groups is presented in File S1.

Additional graphical representation of the abundance differences in DRPs between the cuticle, intestine, and the rest of the body of *A. simplex* s. s. is shown in Figure 2.

Among the proteins found in the intestine (File S1), we identified those with catalytic activity, including carboxypeptidase (A0A0M3K6G9), glutamate dehydrogenase (A0A0M3JI05), fructose-bisphosphate aldolase (A0A0M3JYW9), alanine-glyoxylate aminotransferase (A0A0M3K6B4), or aminopeptidase (A0A3P6SUT5), and proteins that involved both oxidative phosphorylation and the tricarboxylic acid cycle (the processes that generate energy), e.g., oxoglutarate dehydrogenase (A0A0M3JPF6) and succinate dehydrogenase (A0A0M3JRB2).

In the cohort of proteins more abundant in the cuticle of the parasite (File S1), we noticed proteins which take part in the immune response, e.g., galectin (A0A0M3JA06) or twitchin (A0A158PN23); proteins directly involved in the cuticle formation, e.g., the N-terminal region of nematode cuticle collagens (A0A0M3JZM9, A0A0M3IYS8, A0A0M3JZM7) or extracellular matrix proteins, e.g., papilin (A0A0M3KE06) or ADAMTS-like protease (A0A0M3K300); and proteins involved in glycolysis/gluconeogenesis, e.g., cofactor-independent phosphoglycerate mutase (A0A0M3JK61), PEPCK_N domain-containing protein (A0A0M3J768), or pyruvate dehydrogenase E1 component subunit beta (A0A0M3KJM6).

### 2.2. Functional Enrichment Analysis and Pathway Identification

#### 2.2.1. Gene Ontology (GO) Analysis

**Figure 3:**
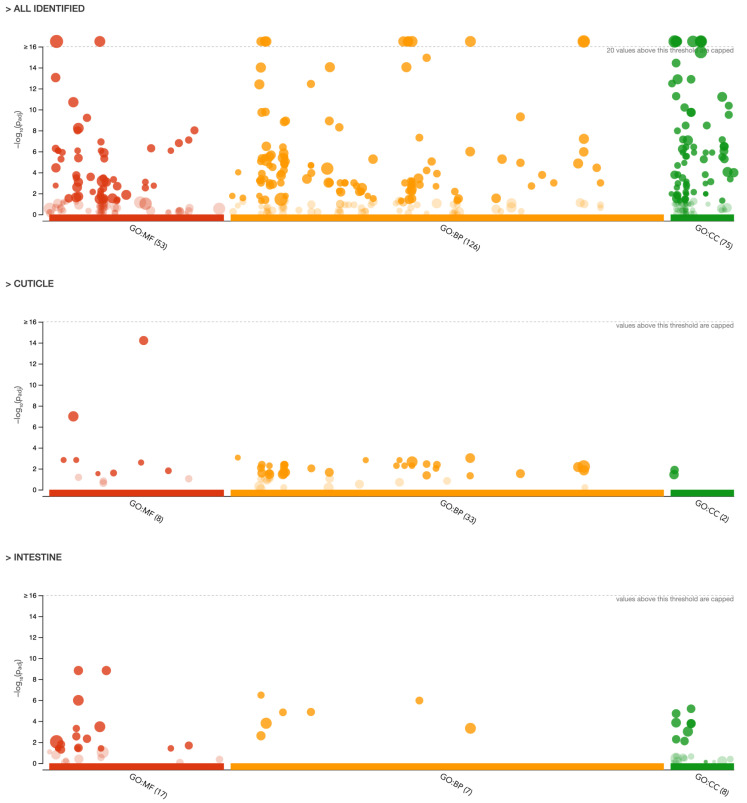
Manhattan plot illustrating the results of the GO analysis. The functional terms are grouped and color-coded by data sources, i.e., molecular function (MF; in red), biological processes (BP; in orange), and cellular components (CC; in green). The five top subcategories from each category are described in Table 1. Detailed representation and annotation of all proteins submitted to the analysis can be found in Files S1 and S2.

Gene ontology analysis assigned all the obtained proteins (*n* = 1831) into 3 different categories: molecular function (MF, 53 different functions), biological processes (BP, 126 different processes), and cellular components (CC, 77 different components) (Figure 3). Detailed annotation can be found in the Supplementary Materials File S2.

Two cohorts of proteins, those more abundant in the intestine and cuticle, were additionally assigned into the GO categories (Figure 3). The top subcategories (a = 0.05) assigned to each of the 3 main GO annotations for the protein cohorts obtained for the intestine and cuticle are shown in Table 1.

Proteins more abundant in the intestine were annotated to the molecular functions (MF category), such as *serine* *hydrolase activity, serine-type peptidase activity,* and *peptidase activity* (GO:0017171, GO:0008236, and GO:0008233, respectively). Moreover, proteins with *oxidoreductase activity* (GO:0016491) were also found in the intestine of *A. simplex* s. s. L4 larvae (Table 1). In the BP category, most of the proteins were involved in the *tricarboxylic acid cycle* (GO:0006099) and *cellular respiration* (GO:0045333). In the cellular component category, most of the proteins found in the intestine were assigned, among others, to the *mitochondrion*, *mitochondrial membrane,* and *mitochondrial envelope* (GO:0005739, GO:0031966, GO: 0005740).

The molecular functions (MF category) assigned to the proteins characteristic of the cuticle were *structural constituent of cuticle* and *structural molecule activity* (GO:0042303 and GO:0005198), *hexokinase activity* and *glucose binding* (GO:0004398 and GO:0005536), and *glutathione hydrolase activity* (GO:0036374) (Table 1). In the BP category, most of the proteins characteristic of the cuticle were involved in the *cellular glucose homeostasis* (GO:0001678), *glucose homeostasis* (GO:0042593), and *carbohydrate homeostasis* (GO:0033500), and in the *nucleobase-containing small molecule metabolic process* and *small molecule metabolic process* (GO:0055086 and GO:0044281) (Figure 3, Table 1). The distribution of the identified proteins according to their abundance in the cellular components was associated with the *extracellular region* (GO:0005576) and *extracellular space* (GO:0005615) (Figure 3, Table 1) (detailed annotation in File S2).

#### 2.2.2. Pathway Identification

**Figure 4:**
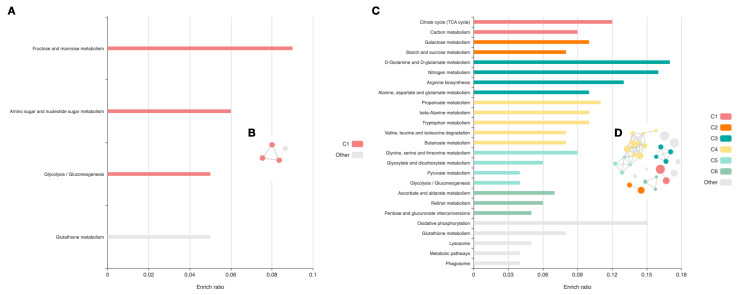
Pathways statistically significantly associated with proteins identified in the cuticle (**A**,**B**) and intestine (**C**,**D**) of the L4 development stage of *Anisakis simplex* s. s. Enriched terms for the cuticle and intestine are visualized in bar plots (**A**,**C**), respectively. Each row represents an enriched function, and the length of the bar represents the enrich ratio, which is calculated as input protein number/background protein number. The color of the bars represents different clusters. For each cluster, if there are more than 5 terms, the top 5 with the highest enrich ratio is shown. Panels (**B**,**D**) are circular network views (cirFunMaps) for the cuticle and intestine, respectively. The node color represents different clusters. Each node represents an enriched term, and node size represents 6 levels of *p*-value, node size from small to large: [0.05, 1], [0.01, 0.05], [0.001, 0.01], [0.0001, 0.001], [1 × 10^−10^, 0.0001], [0, 1 × 10^−10^]; the edge represents interaction larger than the user-defined threshold (0.35). Detailed description of the analysis results can be found in File S3. C1–C6—clusters from 1 to 6.

**Figure 5:**
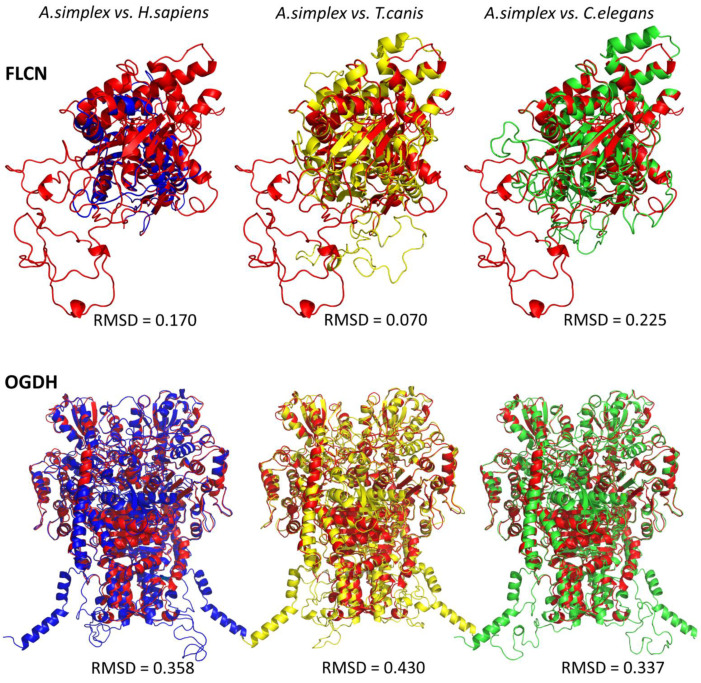
Predicted tertiary structures of *A. simplex* s. s. oxoglutarate dehydrogenase (OGDH) and folliculin (FLCN). The structures are color-coded (structural alignment) for the respective species: *A. simplex* s. s.—red, *H. sapiens*—blue, *T. canis*—yellow, *C. elegans*—green. The root mean square deviation (RMSD) value indicates the average deviation between the corresponding atoms of two proteins: the smaller the RMSD, the more similar the two structures are.

When metabolic pathways were constructed based on the Kyoto Encyclopedia of Genes and Genomes (KEGG), the most abundant proteins in the cuticle were assigned to three metabolic pathways (Figure 4A). The most enriched pathways included *fructose and mannose metabolism* (enrichment ratio = 0.09375, *p*-value = 0.002) and *glycolysis/gluconeogenesis* (enrichment ratio = 0.04672, *p*-value = 0.001). In the cohort of intestinal proteins, analysis revealed involvement in 31 signaling pathways. Those most enriched are shown in Figure 5. Those with the lowest p-value included the *citrate cycle (TCA cycle)* (*p*-value = 6.49 × 10^−9^) and *carbon metabolism* (*p*-value = 1.92 × 10^−11^). The detailed results of the analysis can be found in File S3. Circular network views (cirFunMaps) for the cuticle and intestine are shown in Figure 5, respectively. Each node represents an enriched term, and the color of the node represents different clusters. The maps show the interactions between the clusters and the enriched terms. The *p*-value of the clusters and the size of the clusters can be found in File S3.

### 2.3. Enzymes Identification

The InterPro protein family database was used to identify tissue-specific enzymes from *A. simplex* s. s. L4 larvae. In total, 32 cuticular proteins were assigned to 5 enzyme classes and 66 intestinal proteins were assigned to 6 enzyme classes. The most abundant enzyme class was hydrolases (10 proteins in CUT, 28 in INT), and the less represented classes were oxidoreductases, translocases, and transferases in INT, whereas oxidoreductases, transferases, and lyases in CUT. No proteins belonging to the class of isomerases were detected in INT, and no ligases and translocases were detected in CUT. The distribution of the number of proteins in each enzyme class is shown in File S4.

### 2.4. Tertiary Structure Protein Modeling and Its Detection with Western Blot

Multiple sequence alignment (MSA) was performed to compare the identity of the sequences of the proteins of interest from *A. simplex* s. s., oxoglutarate dehydrogenase (OGDH) and folliculin (FLCN), with the same proteins from *H. sapiens*, *T. canis*, and *C. elegans* (Figure S1; selection criteria explained in the discussion). The structural models of OGDH and FLCN were built for the A0A3P6SZK1 and A0A0M3JT48 sequences, respectively.

The predicted tertiary structures of *A. simplex* s. s. OGDH and FLCN and their structural similarity with the same proteins of other species are shown in Figure 5. The root mean square deviation (RMSD) value indicates the average deviation between the corresponding atoms of two proteins: the smaller the RMSD, the more similar the two structures are. The FLCN of *A. simplex* was structurally most homologous to the FLCN of *T. canis* (RMSD 0.07), less homologous to the human FLCN (RMSD 0.17), and least homologous to the FLCN of *C. elegans* (RMSD 0.225). The 3D structure of OGDH was structurally similarly homologous to human and *C. elegans*, where the RMSD was 0.358 and 0.337, respectively. The OGDH of *A. simplex* s. s. was least homologous to the OGDH of *T. canis* (RMSD 0.430) (Figure 5). Detailed characteristics of all protein sequences subjected to analysis can be found in File S5.

To confirm the presence of OGDH and FLCN in a complex mixture of *A. simplex* s. s. proteins and to validate the 3D structural modelling and LC-MS/MS, Western blot analysis was performed (Figure S2). Two extracts were prepared from two *A. simplex* s. s. stages (L3 and L4). Due to the high sequence similarity of OGDH and FLCN with other species, specific antibodies recognizing endogenous levels of total OGDH and FLCN proteins were used for detection in *A. simplex* s. s. larvae. The same analysis was performed using a porcine protein extract as a positive control. Western blot analyses revealed the presence of an immunoreactive band at 70 and 100 kDa, corresponding to calculated molecular masses of FLCN and OGDH of 72.5 and 90.2 kDa, respectively (Figure S2).

### 2.5. Protein–Protein Interactions (PPIs)

#### 2.5.1. Global PPI Network

**Figure 6:**
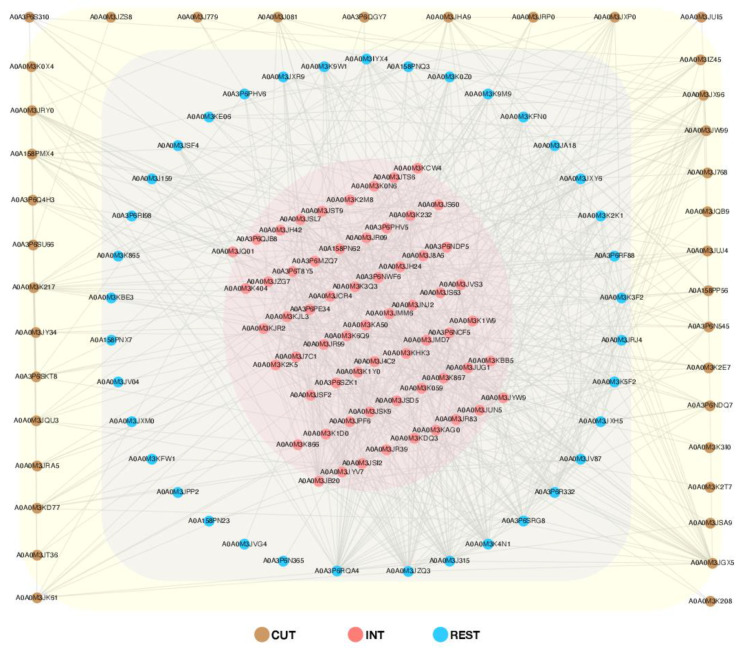
Protein–protein interaction network analysis of differentially regulated proteins between the cuticle, intestine, and rest of the body of the L4 development stage of *Anisakis simplex* s. s. Visualization was performed in Cytoscape v. 3.9.1. A detailed description of all proteins submitted to the analysis can be found in File S6.

The network of protein interactions was performed by submitting only DRPs to the STRING database (v. 11.5.) [30]. All interactions were indicated in the context of co-expression, co-occurrence, and based on the emergence of information about the interactions between these proteins in different databases. The analysis revealed that a total of 136 proteins formed a very complex and highly interactive network (653 interactions) (Figure 6). The proteins characterized for the tissues studied (cuticle and intestine) and the rest of the body of *A. simplex* s. s. were additionally grouped (Figure 6). There were 38 proteins in the cuticle group (233 interactions), 63 proteins in the intestine (496 interactions), and 38 proteins (362 interactions) were observed in the group characterized for the rest of the larval body at the L4 stage (Figure 6). The remaining input proteins, not shown in Figure 6, were excluded. This primarily reflects the fact that these interactions were identified on a nematode *Toxocara canis* background (*A. simplex* not available in the STRING database) and that many of the input proteins were uncharacterized in the Universal Protein Resource (UniProt) [31]. A detailed description of all proteins submitted to the analysis can be found in File S6.

#### 2.5.2. Host–Pathogen Protein Interactions

**Figure 7:**
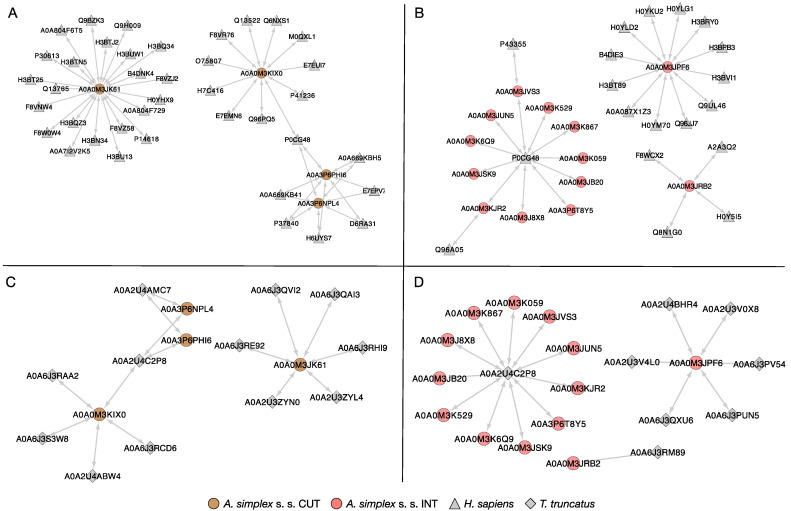
Host–parasite interactions between specific tissues of the L4 development stage of *Anisakis simplex* s. s. and selected hosts. Interactions with the human (*Homo sapiens*) proteome and proteins identified in the cuticle and intestine of *A. simplex* s. s. are presented in part (**A**,**B**), respectively. Interactions with the Atlantic bottlenose dolphin (*T. truncatus*) proteome and proteins identified in the cuticle and intestine of *A. simplex* s. s. are presented in part (**C**,**D**), respectively. Visualization was performed in Cytoscape v. 3.9.1. A detailed description of all proteins submitted to the analysis can be found in File S7.

The HPIDB 3.0 server was used to predict host–parasite interactions between the *A. simplex* s. s. tissue proteins and both human and dolphin proteins. In total, 39 proteins of human and 4 *Anisakis* cuticular proteins were identified in the host–parasite interaction network (Figure 7A). These proteins formed the four main clusters in which the *Anisakis* proteins formed their core: cofactor-independent phosphoglycerate mutase (A0A0M3JK61, 22 interactions), serine/threonine-protein phosphatase (A0A0M3KIX0, 11 interactions), and 2 peptidase M1 domain-containing proteins (A0AP6PHI6 and A0A3P6NPL4, 7 interactions each). Intestinal proteins of *A. simplex* larvae together with human proteins constituted three clusters, among which two parasite proteins formed their core: oxoglutarate dehydrogenase (succinyl-transferring; A0A0M3JPF6, 12 interactions), and succinate dehydrogenase [ubiquinone] iron-sulfur subunit, mitochondrial (A0A0M3JRB2, 4 interactions). One of the formed clusters had as its core a human protein: polyubiquitin-C (P0CG48, 11 interactions) (Figure 7B).

All *Anisakis* tissue-specific proteins identified in this interactomes (Figure 7A,B) were also identified in the dolphin–parasite interaction networks (Figure 7C,D). The same four proteins of the larvae identified in the cuticle were involved in the interactions with proteins of the Atlantic bottlenose dolphin (*Tursiops truncatus*) (Figure 7C); however, the network was less complex (15 interactions in total, compared to 47 in the human–parasite network). The highest number of interactions showed one of the dolphin proteins, polyubiquitin-B isoform X1 (A0A2U4C2P8, 11 interactions). The detailed results of the identification of proteins involved in potential host–parasite interactions are presented in File S7.

### 2.6. Allergens Identification

The AllerCatPro server determined the allergenic properties of cuticular proteins, where 28 had strong evidence and 7 had weak evidence. Among the proteins identified in the intestine, 10 proteins were predicted with strong evidence and 10 with weak evidence to have allergenic potential (File S8). Most of the detected potentially allergic proteins showed high similarity to the proteins of the American house dust mite, *Dermatophagoides farina*. The other detected putative allergens showed similarity to allergens of *Bos taurus*, *Oncorhynchus mykiss*, *Salmo salar*, *Trichostrongylus colubriformis*, and *Aspergillus fumigatus*. The representatives of potential allergens found in the *A. simplex* s. s. tissue-specific proteomes are presented in Table 2.

## 3. Discussion

In accordance with the increasing incidence of anisakiasis, the molecular mechanisms responsible for the pathogenicity of *Anisakis* spp. remain unknown [32]. From a parasitological perspective, the cuticle and intestine are the tissues most responsible for parasite–host contact [33,34] and have long been thought to be involved in such mechanisms through their proteomic composition and, in particular, through the release of excretory-secretory proteins [35,36,37,38]. Moreover, most enzymes important for parasite growth and development are downregulated from the L1 to L3 developmental stages, including enzymes involved in carbohydrate, lipid, and energy metabolism, but many of them are upregulated in L4 and adult nematodes [39,40].

Therefore, in this study, we described for the first time the proteomic analysis of 2 tissues of the L4 developmental stage of *A. simplex* s. s. In the L4 stage, tissues, such as the cuticle and intestine, are fully developed and functional [27,28,29]. In addition, previous proteomic studies of *A. simplex* did not focus on comparative analysis of nematode tissues [41,42,43,44,45,46,47,48,49,50,51]. This work provides new insights into the tissue-specific proteome of *A. simplex* s. s., which was not previously possible. Our knowledge has been limited because it is difficult to isolate individual tissues, especially when the tissue is limited, as in small nematode larvae, and to characterize specific proteome profiles. To identify tissue-specific proteomes in *A. simplex* s. s., we used liquid chromatography-tandem mass spectrometry with tandem mass tag (TMT) labeling, a method that is accurate and capable of measuring small abundance differences in biological samples [52].

The cuticle of nematodes serves as the primary interface with the environment [33], and the proteome composition of the cuticle changes as the nematode organism develops [34,53,54]. The surface of the cuticle of nematodes contains many thermally stable allergens and other catalytic proteins, the presence of which is essential for nematode survival and development in the host [33,34]. The intestine of nematodes is a simple, hallow, straight, non-muscular tube consisting of a single layer of epithelial cells [34]. There is strong evidence that most secretion and excretion occurs through the parasite’s organs, including the excretory gland, esophagus, intestine, and cuticle [54,55,56]. Enzymes that play a fundamental role in metabolism, such as proteases, nucleotidases, esterases, glycases, and dismutases, are associated with infectivity, immune defense, and pathogenicity in parasites, thus playing an extremely important role in the life cycle of parasitic nematodes [33,37,38,53].

Although various approaches have been used to study the biology of nematodes and their interactions with the host, the proteomics of these two tissues—cuticle and intestine at the L4 stage—has not yet been studied. TMT-based LC-MS/MS analysis of L4 larvae of *A. simplex* s. s. allowed the identification of 107 DRPs between the cuticle and the rest of the larval body at the L4 stage, of which 49 were more abundant in the cuticle and 58 in the rest of the nematode body. When comparing the intestine and the rest of the larval body at the L4 stage, 123 proteins were identified as DRPs, with 67 proteins being more abundant in the intestine and 56 in the rest of the *A. simplex* s. s. body. Comparison of the individual tissues examined revealed a total of 272 DRPs, with 133 proteins more abundant in the cuticle and 139 proteins more abundant in the intestine (see Figure 1 and Figure 2, Files S1,4–6). This is currently the largest proteomic dataset of *A. simplex* s. s. tissue-specific proteins. In addition, we again compared the proteomes of L3 and L4 larvae with the most recent reference proteome of *A. simplex* from the UniProt/TrEMBL database (downloaded November 2021; proteome ID: UP000267096). Compared to the results obtained previously by Stryiński et al. [43], 142 DRPs were identified between the L3 and L4 larval stages (see File S9 and Figure S3), with 43 proteins being more abundant at the L3 and 99 proteins at the L4 larval stage. These results complement and update the previously published *A. simplex* proteome [43].

In general, the identified tissue-specific proteins of *A. simplex* s. s. have multiple functions, as shown by analysis of GO. On average, 43 and 32 GO terms were detected for the cuticle and intestine, respectively, for all proteins in each cohort subjected to analysis (see Figure 3, File S2). Annotation enrichment analysis provided interesting results. Of the enriched annotations, the most common and most enriched GO terms among cuticular proteins in general were those related to glucose and carbohydrate homeostasis and cuticle development. These annotations cover functions important for parasite metabolism and survival under anaerobic conditions in the host organism [57]. Of the GO terms annotated for intestinal proteins, most were related to enzymatic activity (hydrolases, peptidases, oxidoreductases) and proteolysis. This is consistent with a previously published study on L4 larvae of *A. simplex*, which found that ligases, lyases, oxidoreductases, and transferases are overrepresented in L4 stage larvae [43]. In addition, enzyme classes associated with cuticular and intestinal proteins, such as hydrolases, and the less represented classes, such as oxidoreductases, translocases, and transferases, in the intestine while oxidoreductases, transferases, and lyases in the cuticle are critical for host–parasite interactions, such as host invasion, migration through host tissues, molting, degradation of hemoglobin and other blood proteins, digestion of nutrients, evasion of the immune system, and activation of inflammation [58,59,60]. Therefore, inhibition of enzymes in these metabolic pathways represents a potential therapeutic strategy to combat parasite infections. However, the high degree of similarity between host and parasite enzymes makes this strategy potentially difficult. Nevertheless, there are several drugs for the treatment of helminth infections that target metabolic enzymes [61,62]. Of particular note are the enzymes involved in glycolysis and the tricarboxylic acid cycle (TCA cycle), as these metabolic pathways were most enriched by cuticular and intestinal proteins of *A. simplex* s. s. L4 stage, respectively (see Figure 4, File S3). Glycolytic and oxidative metabolism in the larvae of *Anisakis* spp. had already been studied by Hamajima et al. but in 1969 [63]. Therefore, it was decided to investigate the obtained results also with regard to the proteins of energy and/or carbohydrate metabolism in *A. simplex* s. s. L4 larvae.

The first step of glycolysis is catalyzed by hexokinase (EC 2.7.1.1). In the group of proteins more abundant in the cuticle, 3 phosphotransferases (A0A3P6NT27, A0A0M3JT86, A0A3P6S264) were found to possess hexokinase activity (see File S4). Studies on *Trypanosoma brucei* have validated this enzyme as a drug target [64]. In this parasitic kinetoplastid, a 60% reduction in cellular hexokinase activity results in parasite death. The hexokinase of *B. malayi*, unlike the monomeric and dimeric forms found in mammals, is tetrameric and has different kinetic parameters than the human enzyme [65,66]. Modified monosaccharides have been proposed as general inhibitors or antimetabolites targeting hexokinase [67]; however, little action has been taken to utilize this proposal with hexokinases of helminth parasites [61]. Phosphoglycerate mutase (PGM) is an enzyme that catalyzes step 8 of glycolysis. These enzymes are classified into 2 different classes: either cofactor-dependent (EC 5.4.2.11, dPGM) or cofactor-independent (EC 5.4.2.12; iPGM) [68]. In most eukaryotes, PGM requires a tightly bound 2,3-bisphospho-D-glycerate cofactor. However, nematodes have an iPGM [69], and such an iPGM was detected in the cuticle of L4 larvae of *A. simplex* (A0A0M3JK61) in this study (see File S4). In addition to the requirement for cofactors, the two types of PGMs also differ in sequence, length, and catalytic mechanisms. For this reason, iPGM has attracted the interest of scientists as a potential drug target. Several potential inhibitors have been identified using computational chemistry methods but have not yet been tested in vitro or in vivo [70]. Recently, studies in *Caenorhabditis elegans* have shown that iPGM is essential for viability [69]. However, a last screening study has shown that iPGM has limited use as a drug [71]. Nevertheless, the anthelmintic Clorsulone can successfully and selectively inhibit the nematode enzyme in *Fasciola hepatica*, which has a host-like dPGM. This suggests that the search for novel molecules that can inhibit the iPGM of *A. simplex* may be the basis for further exploration of methods to control anisakiasis.

The final reaction of glycolysis leading to the second generation of ATP may differ greatly in different helminths [57]. Pyruvate kinase (EC 2.7.1.40; PK) can convert phosphoenolpyruvate (PEP) to pyruvate, which can then be reduced to lactate or ethanol in the cytosol or translocated to the mitochondrion for further oxidation by the TCA cycle. In contrast, phosphoenolpyruvate carboxykinase (EC 4.1.1.32; PEPCK) can carboxylate PEP to oxaloacetate (OAA), an intermediate of the TCA cycle [57,72,73]. This pathway is common in many parasites, especially lumen-dwelling helminths, such as *Ascaris suum* [57]. PK (A0A0M3JXT3) and 3 of 4 PEPCK proteins (A0A0M3IZF7, A0A0M3IZU8, A0A0M3J768) were detected in *A. simplex* tissues in this study but were not found to be statistically significant between the tissues examined (see File S1.4–S6). The fourth PEPCK protein (PEPCK_N domain-containing protein, A0A0M3J768) was more abundant in the cuticle of L4 larvae of *A. simplex* s. s. (see File S1.4–S6). Despite the lack of statistical significance, the abundance of PK ranged from 66 [a.u.] in REST to 188 [a.u.] in INT, where PEPCK levels were approximately 2000–2500 [a.u.] in all examined tissues (see S1.4–6). This is consistent with previous studies on *A. simplex*, where no activity was detected for PK and high activity of PEPCK was observed [74,75]. Moreover, activity of PEPCK increased following molting to the L4 stage [74,75].

If PEP is used to directly generate OAA, which is subsequently reduced to malate (MA) in the life stages of the parasite under anaerobic conditions [61,76,77], the reaction catalyzed by PK is omitted and helminth PKs have been relatively understudied [61]. Thus, since PK is unlikely to be active for the anaerobic steps of the parasitic nematode life cycle, its potential as a drug target may be limited [78,79]. Furthermore, pyruvate can be directly carboxylated to OAA by pyruvate carboxylase (EC 6.4.1.1; PC) [72,73]. This enzyme was detected in the intestine of *A. simplex* s. s. L4 larvae (A0A0M3K059) in this study, which is statistically significant compared to the cuticle (see Files S1.6 and S4). Accordingly, regulation of the PK/PEPCK branch point is potentially important in many helminths and further studies on this topic in *A. simplex* may be based on the results presented.

Glycolysis and the TCA cycle are linked by a reaction catalyzed by pyruvate dehydrogenase complex (PDC), a complex of three enzymes: pyruvate dehydrogenase (EC 1.2.4.1; PDH), dihydrolipoyl transacetylase (EC 2.3.1.12; DLAT), and dihydrolipoyl dehydrogenase (EC 1.8.1.4; DLD), and which converts pyruvate to acetyl-CoA by a process called pyruvate decarboxylation. Acetyl-CoA can then be used in the TCA cycle to carry out cellular respiration [72,73]. In the present study, the abundance of pyruvate dehydrogenase E1 component subunit beta (PDH, A0A0M3KJM6) was found to be much higher in the cuticle than in the intestine (see Files S1.6 and S4). The whole PDC has been studied in detail in *A. suum* [80,81,82,83]. It has been shown that a single phosphorylation catalyzed by pyruvate dehydrogenase kinase (EC 2.7.11.2) can inhibit activation of the PDC in higher eukaryotes, but a greater degree of phosphorylation is required for inactivation of the PCD in *A. suum* [84] Consequently, the PCD remains active to some degree. Nevertheless, to the authors’ knowledge, the PDC has never been studied in *A. simplex*, and this issue requires further investigation. Compared to glycolytic enzymes, TCA enzymes have, generally, received less attention in parasitic nematodes. However, Rahman et al. [85] showed that in *C. elegans* embryos, downregulation of the components of the TCA cycle inhibits entry into the first mitotic division and particularly, knockdown of citrate synthase (EC 2.3.3.1; CS) selectively inhibits the TCA cycle and blocks mitosis [85]. This revealed a previously unknown link between the TCA cycle and cell cycle progression. In the present study, the first enzyme of the TCA cycle, the CS (A0A0M3K529), was detected at significantly higher levels in the intestine than in the cuticle of L4 larvae of *A. simplex* s. s. (see Files S1.6 and S4).

Like the PDC, the oxoglutarate dehydrogenase complex (OGDC), known primarily for its role in the TCA cycle, has also been studied in *Fasciola hepatica* [86]. The OGDC consists of three enzymes: oxoglutarate dehydrogenase (EC 1.2.4.2; OGDH), dihydrolipoyl succinyltransferase (EC 2.3.1.61; DLST), and the previously mentioned DLD (EC 1.8.1.4). Diaz and Komuniecki [86] showed that the enzyme from *F. hepatica* is not upregulated by calcium ions, in contrast to the mammalian OGDH [87,88]. This issue also needs further investigation from the point of view of the effect of inhibitory substances on OGDH to kill helminths. In this study, we found statistically greater amounts of all 3 OGDC enzymes (OGDH, A0A3P6SZK1; DLST, A0A3P6T8Y5; DLD, A0A0M3JB20) in the intestinal tissue of *A. simplex* s. s. compared with the cuticle (see Files S1.6 and S4). OGDH was present in the DRPs cohort under 2 different UniProt IDs (A0A0M3JPF6, A0A3P6SZK1). In addition, we were able to detect the presence of OGDH in *A. simplex* L3 and L4 larvae for the first time using Western blot analysis (see Figure S2). This also prompted us to select OGDH for 3D structural modelling, which is described below. Chin et al. [89] reported that supplementation of α-ketoglutarate, an intermediate of the TCA cycle produced by oxidative decarboxylation from isocitrate and converted to succinyl-CoA by OGDC, delays ageing and prolongs the lifespan of adult *C. elegans*. In addition, increasing the α-ketoglutarate level by RNA interference of *ogdh* also prolongs the lifespan of worms [89]. This may suggest that increasing the activity of OGDC and thus decreasing the α-ketoglutarate level may be lethal to parasites. In addition, α-ketoglutarate can also be produced anaplerotically from glutamate by oxidative deamination using glutamate dehydrogenase (EC 1.4.1.2; GDH) [73]. This enzyme was detected in the intestine of L4 stage larvae of *A. simplex* (A0A0M3JCR4) in this study (see Files S1.6 and S4). The combination of increased OGDC activity and GDH inhibition could potentially lead to the expected results in terms of nematode larval death, but targeted research in this area is needed.

Adult parasitic helminths are not capable of gluconeogenesis. In contrast, gluconeogenesis can function in aerobic larval stages that use substrates other than carbohydrates for energy [57]. However, because L4 larvae are more similar to adults than L3 larvae, it was decided not to discuss gluconeogenesis.

In the current study, we also decided to take a closer look at folliculin (FLCN) as an inhibitor of lactate dehydrogenase A (EC 1.1.1.27; LHDA) and as regulator of the Warburg effect [90]. FLCN was selected for further study based on the PPI analysis results (see File S6). The sequence of the thioredoxin domain-containing protein (A0A0M3JRA5) of *A. simplex* s. s. (very abundant in the cuticle) resembled the FLCN of *T. canis* (A0A0B2VPH2), whose proteome formed the background for the PPI analysis. After searching the UniProt database, it was found that the FLCN protein for *A. simplex* s. s. was already annotated (A0A0M3JT48). However, LC-MS/MS analysis did not reveal the presence of this protein in the tissues studied but only the similar protein containing a thioredoxin domain (A0A0M3JRA5). Therefore, it was decided to compare the 2 *A. simplex* s. s. sequences (A0A0M3JRA5, A0A0M3JT48) and, like in case of OGDH, build a 3D model of the FLCN protein of *A. simplex* s. s. on this basis.

FLCN acts as a binding partner and non-competitive inhibitor of LDHA [90]. LDHA converts pyruvate, the end product of glycolysis, to lactate in the absence of oxygen [72]. The presence of LDH was noted in many helminth species as early as 1967 [91]. LDH activity is common in parasitic nematodes and removes pyruvate produced by PK that is not passed to the mitochondria. It is also thought to be involved in the regulation of the redox balance in the cytosol of helminth cells [92]. In the current study, the 2 forms of lactate dehydrogenase (LDHD and LDHL), in which L-lactate dehydrogenase plays a key role in the production of lactate from pyruvate, were identified in *A. simplex* s. s. L4 larvae (A0A0M3KCR5 and A0A3P6PI01) (see File S1.2). In addition, we confirmed for the first time the presence of FLCN in *A. simplex* s. s. L3 and L4 larvae by Western blot analysis (see Figure S2).

A flexible loop in the amino terminus of FLCN controls the movement of the loop in the active site of LDHA, regulating enzyme activity and consequently metabolic homeostasis in normal cells. Cancer cells in which the Warburg effect occurs show dissociation of FLCN from LDHA. Treatment of these cells with a decapeptide from the FLCN loop region results in cell death. The glycolytic shift of cancer cells appears to be the result of FLCN inactivation or dissociation from LDHA [90]. Although the Warburg effect has not been studied in the context of helminth metabolism often, it is worth mentioning with respect to FLCN and lactate production.

The Warburg effect is a metabolic response to an increase in glycolytic flux due to hypoxia or a strictly anaerobic environment [93]. It is also referred to as aerobic glycolysis and is rather inefficient regarding ATP production (~4 mol ATP/mol glucose) when compared to the TCA cycle (~36 mol ATP/mol glucose) [94], which is the primary aerobic metabolism of parasitic larvae (L1-L3) [57]. However, anaerobic glycolysis produces at most ~2 mol ATP/mol glucose, which could explain the metabolic advantage of the larval Warburg effect during the transition to L4 or adult. This postulate has also been mentioned in metabolomic studies of *H. contortus* [95]. In addition, Łopieńska-Biernat et al. [96,97] indicate that the trehalose and glycogen synthesis and degradation pathways play a crucial role in parasitic nematodes’ development and that genes related to these pathways are upregulated in L4 larvae of *A. simplex* s. l. Interestingly, the major pathway of lactate production is thought to be directly linked to trehalose synthesis and degradation [98,99,100]. Consistent with all this, FLCN-mediated inhibition of LDHA hyperactivity during the Warburg effect provides a new paradigm for controlling parasite survival. However, further evidence is needed to investigate such hypotheses in gastrointestinal nematodes and to shed light on the potential role of this alternative metabolic pathway.

**Figure 8:**
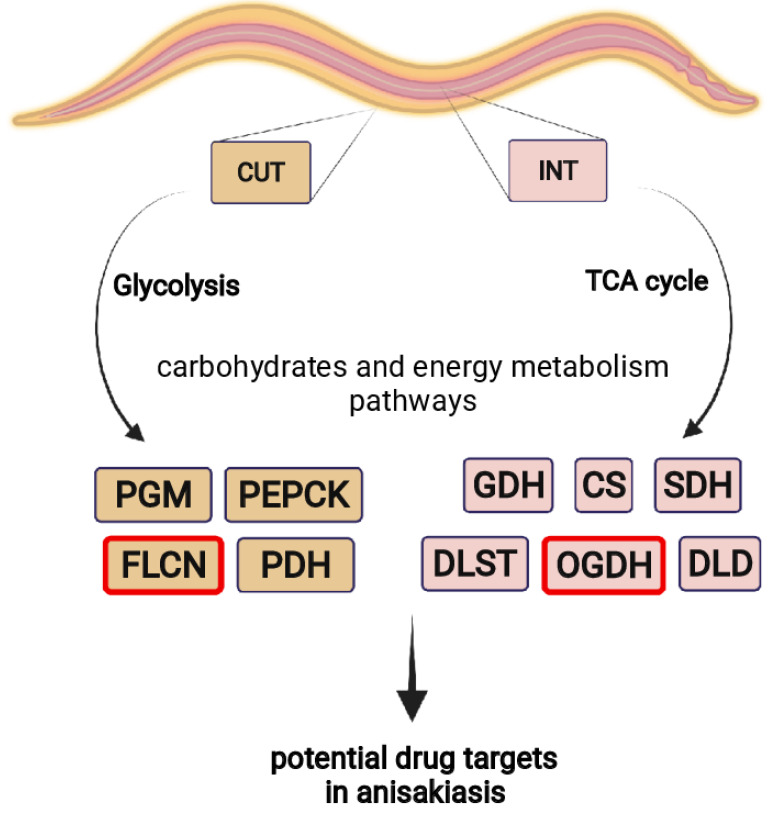
Summary of the results discussed. The possibility of using enzymes of energy and carbohydrate metabolism as potential drug targets in anisakiasis.

The results discussed open new possibilities in the study of enzymes of energy and carbohydrate metabolism as potential drug targets in anisakiasis. Therefore, in vitro or in vivo experiments followed by activity assays are needed to verify the inhibition of the key proteins described in this study (Figure 8).

In addition, the current study provided new information on 3D models of OGDH and FLCN as proteins that may be potential candidates for controlling *A. simplex* survival. The models for OGDH and FLCN were predicted based on the sequences A0A03PSZK1 and A0A0M3JT48, respectively, and the obtained 3D structures were compared with those of other species (see Figure 5). As expected, the tertiary structure of FLCN was very similar to the structure of FLCN from *T. canis*, a parasitic nematode of canids. In the case of OGDH, the situation was reversed. The model showed the greatest structural similarity to OGDH from *C. elegans*. Importantly, there were differences in the structure of *A. simplex* and human proteins (see Figure 6). This could be the basis for further research to select an efficient method to kill nematodes without harming the host (Figure 8).

In addition to the results already discussed (Figure 8), analyses of protein–protein interactions were also performed in the present work. The global PPI network was visualized using the DRPs between the tissues tested (see Figure 6, File S6). The proteins in the presented network interact with an average of 10 other proteins. For example, OGDH interacts with 11 other proteins. Such connections in the case of gene silencing or direct blocking of the activity of a particular protein offer the chance of promising results, as they affect many metabolic pathways simultaneously, which has been discussed previously [41,42,43]. In addition, for the first time for *A. simplex* s. s., the tissue-specific host–pathogen interactions were investigated (see Figure 7, File S7). Interestingly, the parasitic proteins characterized in detail above, such as OGDH, DLST, DLD, PC, or CS, were directly involved in the interactions with the human host. OGDH from the parasite was particularly interactive, and polyubiquitin-C (P0CG48) was the major identified target interacting with *A. simplex* proteins. Ubiquitin is known to modulate host–pathogen interactions, with a particular focus on host innate immune defenses and pathogen immune evasion [101]. This is consistent with the findings of Kochanowski et al. [51], who studied the excretory-secretory proteins of *A. simplex* larvae. The number of proteins involved in the human–parasite interaction network is about twice the number of proteins in the dolphin–parasite interactome. This result could be mainly due to the fact that the available database for non-human host–pathogen interactions is much more limited than for human–pathogen interactions, which was also highlighted by Kochanowski et al. [51]. Cuticular proteins of *A. simplex* interacting with the human or dolphin host included serine/threonine protein phosphatase (A0A0M3KIX0; EC 3.1.3.16; STP), 2 peptidase M1 N-domain-containing proteins (A0A3P6NPL4 and A0A3P6PHI6), and the aforementioned iPGM (A0A0M3JK61) (see Figure 7, File S7). It is worth noting that STPs are integral components of parasitic excretory/secretory proteomes that are thought to be released during host–parasite interactions. Recent studies on *Haemonchus contortus* have shown that STP 1 is actively involved in suppressive regulation of the immune functions of goat peripheral blood mononuclear cells [94]. A group of intestinal proteins that interact with the hosts studied includes the iron-sulphur subunit of succinate dehydrogenase [ubiquinone] (A0A0M3JRB2; EC 1.3.5.1; SDH-IP) (see Figure 7, File S7). Iron-sulphur protein (IP) is an essential component of the mitochondrial complex II (succinate dehydrogenase, SDH), which is a functional enzyme of both the TCA cycle and the respiratory electron transport chain (ETC). SHD-IP is responsible for the transfer of electrons from succinate to ubiquinone (UQ) in normoxia (normal oxygen conditions). Under hypoxia, helminths use fumarate instead of oxygen as the final electron acceptor of ETC [102,103]. This is achieved by an alternative ETC in which rhodoquinone (RQ) serves as an electron carrier [61,102,103,104]. RQ is a redox-active quinone that is structurally similar to conventional UQ [105]. This change confers RQ a lower redox potential than UQ, which allows RQ to accept electrons from reduced nicotinamide adenine dinucleotide (NADH) via complex I and to reduce fumarate to succinate through complex II. In this alternative ETC, complex II functions as a fumarate reductase (FRD), in reverse to the conventional ETC, in which complex II functions as an SDH [104,106]. Mitochondria, especially in the aerobic developmental stages, such as L2 and L3, contain mainly UQ. In contrast, the mitochondria of adult worms living in a low-oxygen environment contain only RQ [107,108,109].

The association of an alternative complex II with RQ is well established in *A. suum* and has become a paradigm in helminth biochemistry. However, the results presented by Otero et al. [106] show that *A. suum* is the exception rather than the rule. ETC complexes appear to have evolved to adapt to different stages of the helminth life cycle, to environmental conditions, or to specific cells or tissues within the worms [106]. To the authors’ knowledge, no studies have been performed on the SDH properties of L4 larvae of *A. simplex*. Moreover, the involvement of UQ vs. RQ at ETC in *A. simplex* has never been directly tested. Perhaps this is why the results discussed above refer to typically anaerobic conditions in L4 larvae and the occurrence of SDH oxidizing succinate to fumarate and reducing UQ may be an unexplored hypothesis so far.

Studies have shown that the parasite SDH enzyme could be a potential target for selective chemotherapeutic agents [110,111]. However, its potential use in *A. simplex* remains to be investigated.

The last analysis performed on proteins characteristic of the intestine and cuticle of *A. simplex* L4 larvae was able to identify allergens and potential allergens. Of the 39 *A. simplex* allergens listed in the ALLERGOME database (available online: https://www.allergome.org, accessed on 17 February 2022), only Ani s 24 kD resembled one of the uncharacterized proteins identified in the cuticle of *A. simplex* in the present study (see Table 2; File S8). In addition, nine potential allergens were also identified in this study (see Table 2; File S8). One of these, fructose-bisphosphate aldolase (A0A0M3JYW9) was also identified as a potential allergen in the secretome of *A. simplex* by Kochanowski et al. [51]. In addition, many of the potential allergens detected in *Anisakis* tissues show similarity to the potential allergens identified by other authors in the whole proteome and transcriptome of *Anisakis* larvae [9,43,45,48,50].

To sum up, the tissue-specific proteomes of the intestine and cuticle of L4 stage larvae of *A. simplex* s. s. were characterized for the first time. Comparison of the individual tissues revealed a total of 272 DRPs, with 133 proteins being more abundant in the cuticle and 139 proteins in the intestine. In general, the identified tissue-specific proteins of *A. simplex* s. s. have multiple functions, as shown by the analyses of GO and KEGG. In addition, host–pathogen interactions were identified, and potential new allergens were predicted. Of particular note are enzymes involved in glycolysis and the TCA cycle, as these metabolic pathways were most enriched by cuticular and intestinal proteins of *A. simplex* s. s. L4, respectively. Therefore, key enzymes and other proteins in the above metabolic pathways were discussed. Beyond iPGM, PK, PEPCK, PDH, CS, DLST, DLD, or GDH, two proteins, OGDH and FLCN, were the focus of our attention. Their presence in L3 and L4 larvae was also confirmed by Western blot, and their tertiary structure was modulated and compared with other species.

The identified tissue-specific proteins, i.e., PGM, PEPCK, GDH, CS, SDH, DLST, DLD, OGDH, and FLCN, could serve as targets for new drugs against anisakiasis. However, it should be noted that the functional analysis of the identified tissue-specific proteins was performed using an omic approach. Therefore, in vitro or in vivo experiments and subsequent activity assays are required to verify the inhibition of the key proteins described in this study. Such a combination of global omic analysis with classical biochemical methods and molecular biology will provide a clear picture of the active metabolic pathways in the biological systems under investigation.

## 4. Materials and Methods

### 4.1. Anisakis Simplex

**Figure 9:**
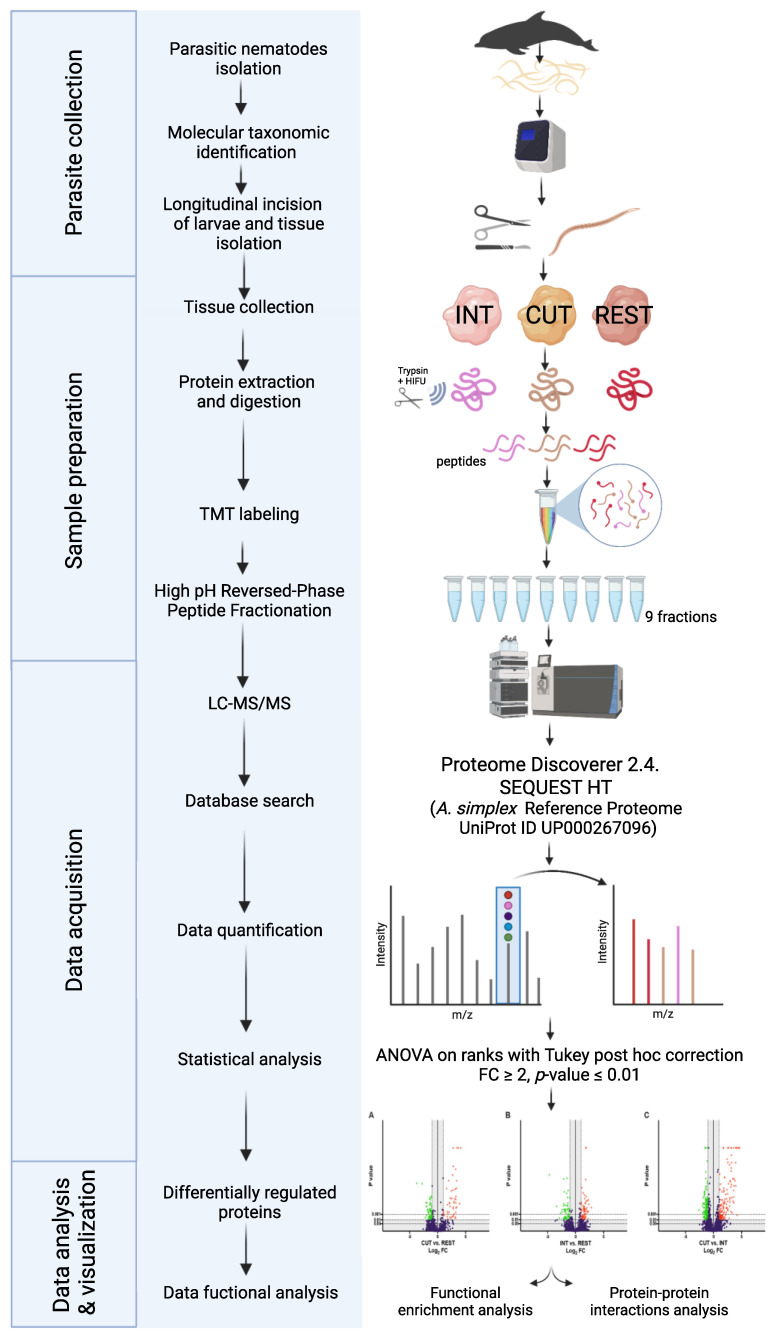
Experimental setup and workflow for comparison of tissue-specific proteomes of *Anisakis simplex* s. s. larvae at the L4 stage. In total, 12 *A. simplex* s. s L4 stage larvae were isolated from striped dolphin (*Stenella coeruleoalba*). Samples of the cuticle, intestine, and remaining body of the L4 larvae were collected; proteins extracted; and subjected to trypsin digestion stimulated with high-intensity focused ultrasound (HIFU). Peptides from each sample were labeled with tandem mass tags (TMTs). After tagging, samples were pooled and fractionated using the high pH reversed phase fractionation method. Tandem mass spectrometry data were acquired using an LTQ-Orbitrap Elite spectrometer and proteins were identified using Proteome Discoverer 2.4. The identified proteins were subjected to statistical analysis and obtained results were visualized. Created with BioRender.com.

The experimental setup and workflow for comparison of tissue-specific proteomes of *Anisakis simplex* s. s. larvae at the L4 stage is presented in Figure 9. All experiments were performed on the L4 larvae of *A. simplex* s. s. isolated from striped dolphin (*Stenella coeruleoalba*). All larvae were obtained from the biobank of the PARASITE project [112] and were previously taxonomically identified using conventional polymerase chain reaction (PCR) to amplify the mitochondrial cytochrome c oxidase subunit II (mtDNA *cox2*) gene, and the elongation factor *EF1* α-1 nuclear DNA gene as described before by Levsen et al. [113] and using microscopical observation of morphological features characteristic of L4 stage larvae.

### 4.2. Sample Preparation

Twelve *A. simplex* s. s. L4 larvae were used in the study. Three whole larvae were placed in sterile test tube and stored at −80 °C until further analysis. The longitudinal incision of the next 9 larvae using a surgical scalpel was performed under the microscope (magnitude × 100). Then, using microscopic tweezers, the outer layer of the larvae—the cuticle—was separated from the body. The cuticle separated in this way was placed in sterile test tubes. A cuticle from three larvae was used for one sample (three samples in total). Then, the intestine of the larvae was isolated, cleaned of any other tissue residues, and transferred to sterile test tubes. An intestine from three larvae was used for one sample (three samples in total). The remains of the body (after cuticle and intestine isolation) of all used larvae were collected in the test tube and further processed. Additionally, 5 L3 stage larvae isolated from hake (*Merluccius merluccius*) were used to compare the global proteomes of L3 and L4 stages (File S3). The taxonomical identification of L3 larvae was performed as described in Section 4.1.

### 4.3. Protein Extraction, Preparation, and LC-MS/MS Analysis

#### 4.3.1. Protein Extraction

Proteins were extracted as described before by Stryiński et al. [114]. The whole larvae and isolated tissues (L3, L4, cuticle sample further as CUT, intestine sample, abbreviated further as INT, and the remains of the rest of the body abbreviated further as REST) were crushed manually with a sterile plastic pestle in 2 mL centrifuge tubes. Then, protein extraction was performed in 1.5 mL of lysis buffer: 60 mM Tris-HCl pH 7.5, 1% lauryl-maltoside (cat. no 89902, Sigma Aldrich, Poznań, Poland), 5 mM PMFS (cat. no 36978, Thermo Fisher Scientific, Waltham, MA, USA), and 1% DTT (cat. no D9779, Sigma Aldrich, Poznań, Poland). The protein concentration was quantified using the bicinchoninic acid method (Pierce BCA Protein Assay Kit, Thermo Fisher Scientific, Waltham, MA, USA) according to the manufacturer’s protocol. A total of 100 μg of the protein from each sample (9 samples in total) were transferred into a new tube and methanol/chloroform precipitation was performed as described by Carrera et al. [115]. Then, ultrafast tryptic digestion with the simultaneous application of high-intensity focused ultrasound (HIFU) was carried out, as described previously by Stryiński et al. [43,114].

#### 4.3.2. TMT Labeling and Reversed-Phase Fractionation

The TMT 10-plex isobaric label reagents (0.8 mg, Thermo Fisher Scientific, Waltham, MA, USA) were resuspended in 41 μL of anhydrous acetonitrile and added to 100 μg of protein digest, as described by Stryiński et al. [114]. Within the experiment, samples were labeled with TMT10-plex as follows (INT: 127C, 128N, 128C; CUT: 129N, 129C, 130N; REST: 130C). Additionally, whole L3 and L4 larvae were labeled (L3: 126; L4: 127N) and submitted to analysis. Samples were combined in a new tube at equal amounts according to the manufacturer’s instructions. The TMT-labeled peptide concentration was measured using a Pierce Quantitative Colorimetric Peptide Assay (Thermo Fisher Scientific, Waltham, MA, USA) according to the manufacturer’s instructions. To increase the number of peptide identifications, eliminate the interference from co-isolated ions, and achieve results comparable to the MS3-based methods [42,116], the combined sample was fractionated using a Pierce High-pH Reversed-Phase Peptide Fractionation Kit (Thermo Fisher Scientific, Waltham, MA, USA) following the manufacturer’s instructions. The peptide concentration in each fraction was determined by colorimetric analysis using the Quantitative Colorimetric Peptide Assay (Thermo Fisher Scientific, Waltham, MA, USA) following the manufacturer’s instructions. Then, fractions were evaporated to dryness using vacuum centrifugation (SpeedVac concentrator, Thermo Fisher Scientific, Waltham, MA, USA). The samples (8 fractions) were stored at −80 °C until further analysis.

#### 4.3.3. LC-MS/MS Analysis and Data Processing

Peptide fractions were acidified with 0.1% formic acid and analyzed by LC-MS/MS using a Proxeon EASY-nLC II liquid chromatography system (Thermo Fisher Scientific, Waltham, MA, USA) coupled to an LTQ-Orbitrap Elite mass spectrometer (Thermo Fisher Scientific, Waltham, MA, USA). Peptide separation (1 μg) was performed as described by Stryiński et al. [43,114]. All acquired MS/MS spectra were analyzed using SEQUEST-HT (Proteome Discoverer 2.4 package; Thermo Fisher Scientific, Waltham, MA, USA) against a reference proteome of *A. simplex* available in the UniProt/TrEMBL database (downloaded November 2021; proteome ID: UP000267096; # of entries 20,779). The following restrictions were used: full tryptic cleavage with up to 2 missed cleavage sites and tolerances of 10 ppm for parent ions and 0.06 Da for MS/MS fragment ions. TMT labeling (+229.163 Da on N-termini and lysine residues) and carbamidomethylation of cysteine (+57.021 Da) were set as fixed modifications. The permissible variable modifications were methionine oxidation (+15.994 Da), acetylation (+42.011 Da) of the N terminus of the protein, and deamidation (+0.984 Da) of asparagine and glutamine. Moreover, searching parameters included four maximal dynamic modification sites.

#### 4.3.4. Statistical Analysis

The results were subjected to statistical analysis to determine the peptide false discovery rate (FDR) using a decoy database and the Target Decoy PSM Validator algorithm [117]. The FDR was kept below 1%, and for further analysis, only proteins meeting selected criteria were submitted: (a) proteins classified as master proteins, (b) proteins with at least 2 unique peptides, and (c) characterized proteins. Relative quantification was performed using the Quantification Mode and normalization against the total peptide amount (Proteome Discoverer 2.4 package, Thermo Fisher Scientific, Waltham, MA, USA). After relative quantification, several filters were applied to obtain the final list of differentially regulated proteins (DRPs): (a) at least a 2-fold change (FC ≥ 2) in normalized ratios, (b) 1-way ANOVA on ranks and Tukey HSD post-hoc test (*p*-value ≤ 0.01).

### 4.4. Results Analysis

#### 4.4.1. Functional Enrichment Analysis and Pathway Identification

To perform functional enrichment analysis, the proteins identified in the cuticle, intestine, and rest of the body of L4 stage larvae of *A. simplex* s. s. were classified into 3 different categories of Gene Ontology (GO): biological processes, cell components, and molecular functions. GO analysis was performed using g:GOSt, the core of the g:Profiler (ELIXIR, Hinxton, Cambridgeshire, UK) that performs statistical enrichment analysis [118] (https://biit.cs.ut.ee/gprofiler/gost, accessed on 22 January 2022). The g:GOSt web-based tool applied an overrepresentation test controlled with the g:SCS algorithm. The significantly enriched functional GO categories were reported by comparing the input data with the background of GO annotations for parasite-specific data from WormBase ParaSite (*Anisakis simplex* PRJEB496).

KEGG pathways in which proteins of each tissue were involved were detected using the KOBAS 3.0 server (http://kobas.cbi.pku.edu.cn**/**, accessed on 19 February 2022) [119]. Enriched KEGG pathways were identified by Fisher’s exact test using the *Brugia malayi* proteome as a reference and the whole *A. simplex* proteome as background. In this case, a *p*-value less than 0.05 with Benjamini and Hochberg correction was considered to indicate significance.

#### 4.4.2. Enzymes Identification

InterPro protein family classification and enzyme identification were performed using OmicsBox with the Blast2GO algorithm (ver. 1.4.12; BioBam Bioinformatics SL, Valencia, Spain) with the default settings [120].

#### 4.4.3. 3D Structures Modulation

The alignment of sequences of interest was performed using the MUSCLE v. 3.8.425 [121]. To approximate an accurate tertiary model of 2 selected *A. simplex* proteins: OGDH and FLCN, we used several protein structure prediction servers, namely FOLDpro [122], I-TASSER [123], 3DJigsaw [124], LOOPP [125], Phyre2 [126], and SwissModel [127]. To assess the quality of the output models and to choose the top candidates, we used Resprox [128], Qmean [126], and ModFOLD [129]. We then manually inspected the top three models of each protein to determine any unresolved secondary structures (i.e., α-helices) and optimize the structures (e.g., by removing steric clashes). The top model structures with an optimized structure were presented. Root mean square deviation (RMSD) was used to measure the average distances between atoms from two protein structures: one based on the *A. simplex* s. s. sequence and the second based on the sequence of *H. sapiens*, *T. canis*, and *C. elegans*, respectively.

#### 4.4.4. Western Blot of Selected Proteins

To confirm the results of the analysis of 3D structures, we performed Western blot analysis for OGDH and FLCN proteins. Samples (L3 and L4 larvae) for Western blot analysis were homogenized on ice with T-PER Tissue Protein Extraction Reagent (Thermo Fisher Scientific, Waltham, MA, USA) enriched with protease inhibitors (Sigma Aldrich, Poznań, Poland). Lysates were clarified by centrifugation (10,000× *g*, 5 min, 4 °C). The protein concentration was quantified using the bicinchoninic acid method (Pierce BCA Protein Assay Kit, Thermo Fisher Scientific, Waltham, MA, USA) according to the manufacturer’s protocol. Western blot analysis was performed according to Smolinska et al. [130]. In total, 30 μg of total protein isolates were dissolved in sample buffer (100 mM Tris-HCl, 4% SDS, 20% glycerol, 0.2% bromophenol blue, and 200 mM dithiothreitol, pH = 6.8) and incubated at 99.9 °C for 5 min. Samples were then separated by SDS-PAGE electrophoresis in a 12.5% polyacrylamide gel (100 V) and transferred to a polyvinylidene fluoride (PVDF) membrane (Roche Diagnostics, Poland). Then, 5% bovine serum albumin (BSA) in Tris-buffered saline containing 0.1% Tween 20 detergent (TBST) was used to block nonspecific binding. Membranes were then incubated at 4 °C overnight with specific primary antibodies: OGDH (1:1000; cat. no 26865; Cell Signaling Technology, Danvers, MA, USA) and FLCN (1:1000; cat. no 3697; Cell Signaling Technology, Danvers, MA, USA). After incubation, the membranes were washed 3 times for 5 min in TBST buffer and then incubated for 1.5 h at room temperature with goat anti-rabbit IgG secondary antibody (AP156P; Merck Millipore, Kenilworth, NJ, USA) conjugated with horseradish peroxidase (HRP). Immunocomplexes were visualized using Immobilon Western Chemiluminescent HRP Substrate (Merck Millipore, Kenilworth, NJ, USA) according to the manufacturer’s protocol and were visualized using the Azure 280 Imaging System (Azure Biosystems, Dublin, CA, USA).

#### 4.4.5. Protein–Protein Interactions Analysis

The PPI analysis was performed by submitting the DRPs dataset to STRING v. 11.5 (ELIXIR, Hinxton, Cambridgeshire, UK, accessed on 22 January 2022) [30,131] and then visualized with the use of Cytoscape v. 3.9.1 (NIGMS, Bethesda, MD, USA), a software for visualizing complex networks [132]. Interactions were identified through comparison of the input data with the background of the *Toxocara canis,* the phylogenetically closest parasitic nematode with the highest number of proteins available in the STRING database. The network was limited only to the proteins that had at least one interaction with other proteins submitted to the analysis.

The host–parasite protein interactions were predicted using the HPIDB 3.0 server (https://hpidb.igbb.msstate.edu/hpi30_index.html, accessed on 21 January 2022) [133,134], which was run with the default setting using *A. simplex* s. s. tissue-specific proteins searched against human (*Homo sapiens*, UniProt proteome ID: UP000005640; # of entries 79,052) and Atlantic bottle-nosed dolphin (*Tursiops truncatus*, UniProt proteome ID: UP000245320; # of entries 45,130) proteomes. Interactome networks were visualized and analyzed using Cytoscape v. 3.9.1 (NIGMS, Bethesda, MD, USA) [132].

#### 4.4.6. Allergen Identification

The protein allergenicity potential prediction was performed with the use of the AllerCatPro server with default settings (v. 2.0; https://allercatpro.bii.a-star.edu.sg/, accessed on 17 February 2022) [135].

## Figures and Tables

**Table 1:** The top subcategories from each GO category obtained in g:Profiler. The adjusted enrichment *p*-values with the number of proteins assigned to each subcategory are presented. For graphical representation, see Figure 4. Detailed annotation of all proteins submitted to the analysis can be found in Files S1 and S2.

Term Category *	Term Name	Term ID	Adjusted *p*-Value	Term Size	Input Data Size
INTESTINE					
MF	serine hydrolase activity	GO:0017171	1.495 × 10^−9^	115	15
MF	serine-type peptidase activity	GO:0008236	1.495 × 10^−9^	115	15
MF	peptidase activity	GO:0008233	1.038 × 10^−6^	417	22
MF	oxidoreductase activity	GO:0016491	3.416 × 10^−4^	488	20
MF	calcium-dependent phospholipid binding	GO:0005544	4.901 × 10^−4^	19	5
BP	tricarboxylic acid cycle	GO: 0006099	3.291 × 10^−7^	18	7
BP	cellular respiration	GO:0045333	1.083 × 10^−6^	32	8
BP	energy derivation by oxidation of organic compounds	GO:0015980	1.338 × 10^−6^	43	8
BP	aerobic respiration	GO:0009060	1.446 × 10^−5^	29	7
BP	proteolysis	GO:0006508	1.596 × 10^−4^	598	23
CC	mitochondrial membrane	GO:0031966	6.598 × 10^−6^	64	8
CC	mitochondrial envelope	GO:0005740	1.882 × 10^−5^	73	8
CC	mitochondrion	GO:0005739	1.402 × 10^−4^	165	10
CC	organelle envelope	GO:0031967	1.713 × 10^−4^	97	8
CC	envelope	GO:0031975	1.713 × 10^−4^	97	8
CUTICLE					
MF	structural constituent of cuticle	GO:0042302	6.155 × 10^−15^	103	16
MF	structural molecule activity	GO:0005198	1.010 × 10^−7^	296	16
MF	hexokinase activity	GO:0004396	1.472 × 10^−3^	6	3
MF	glucose binding	GO:0005536	1.472 × 10^−3^	6	3
MF	glutathione hydrolase activity	GO:0036374	2.562 × 10^−3^	7	3
BP	cellular glucose homeostasis	GO:0001678	8.682 × 10^−4^	6	3
BP	nucleobase-containing small molecule metabolic process	GO:0055086	9.613 × 10^−4^	173	8
BP	glucose homeostasis	GO:0042593	1.513 × 10^−3^	7	3
BP	carbohydrate homeostasis	GO:0033500	1.513 × 10^−3^	7	3
BP	small molecule metabolic process	GO:0044281	2.182 × 10^−3^	497	12
CC	extracellular region	GO:0005576	3.644 × 10^−2^	130	5
CC	extracellular space	GO:0005615	1.324 × 10^−2^	56	4

* MF—molecular function, BP—biological processes, CC—cellular component.

**Table 2:** Five top strong evidence results of the identification of potential allergens in the tissue-specific proteome of *A. simplex* s. s. L4 larvae. Detailed annotation of all proteins submitted to the analysis can be found in File S8.

*Anisakis Tissue-Proteome*			*AllerCatPro Prediction*		
Tissue	UniProt Accession	Protein Name	UniProt/NCBI Accession	Protein Name	Organism
Intestine	A0A3P6Q2Z8	Uncharacterized protein	L7UZ85	Der f 24	*Dermatophagoides farinae*
Intestine	A0A0M3JS03	60S acidic ribosomal protein P2	Q9UUZ6	Asp f 8	*Aspergillus fumigatus*
Intestine	A0A0M3K232	Voltage-dependent anion-selective channel protein 3	Q1HR57	Aed a 6	*Aedes aegypti*
Intestine	A0A0M3JYW9	Fructose-bisphosphate aldolase	B5DGM7	Sal s 3	*Salmo salar*
Intestine	A0A0M3KBB5	Gelsolin-like protein 1	Q8MVU3	Der f 16	*Dermatophagoides farinae*
Cuticle	A0A0M3K208	Uncharacterized protein	G1FMP3	Ani s 24 kD	*Anisakis simplex*
Cuticle	A0A3P6N545	Uncharacterized protein	P59704	Aspartyl protease inhibitor	*Trichostrongylus colubriformis*
Cuticle	A0A158PPI4	Uncharacterized protein	E1BI98	Bos d Collagen 140 kD	*Bos taurus*
Cuticle	A0A0M3JZM7	Col_cuticle_N domain-containing protein	P02465	Bos d alpha2I	*Bos taurus*
Cuticle	A0A0M3JZS8	Uncharacterized protein	O93484	Onc m alpha2I	*Oncorhynchus mykiss*

## Data Availability

The raw data files from LC-MS/MS (Tissue-specific proteome of L4 larvae of *Anisakis simplex* s. s.; #MSV000088491) are in the public domain and freely available through the MassIVE Repository (https://massive.ucsd.edu/ProteoSAFe/static/massive.jsp, accessed on 24 March 2022). Other data results are available upon reasonable request to the corresponding author.

## Supplementary Materials

The following supporting information can be downloaded at: https://www.mdpi.com/article/10.3390/ijms23084336/s1.
