# Supplementary material for: Tandem Mass Tagging (TMT) Reveals Tissue-Specific Proteome of L4 Larvae of Anisakis simplex s. s.: Enzymes of Energy and/or Carbohydrate Metabolism as Potential Drug Targets in Anisakiasis
Source: Int J Mol Sci. 2022 Apr 14;23(8):4336. doi: 10.3390/ijms23084336 (PMC9027741; doi:10.3390/ijms23084336)
Supplement: Supplementary file 1 [file ijms-23-04336-s001.zip › ijms-1675296-Supplementary/Figure S1.pdf]

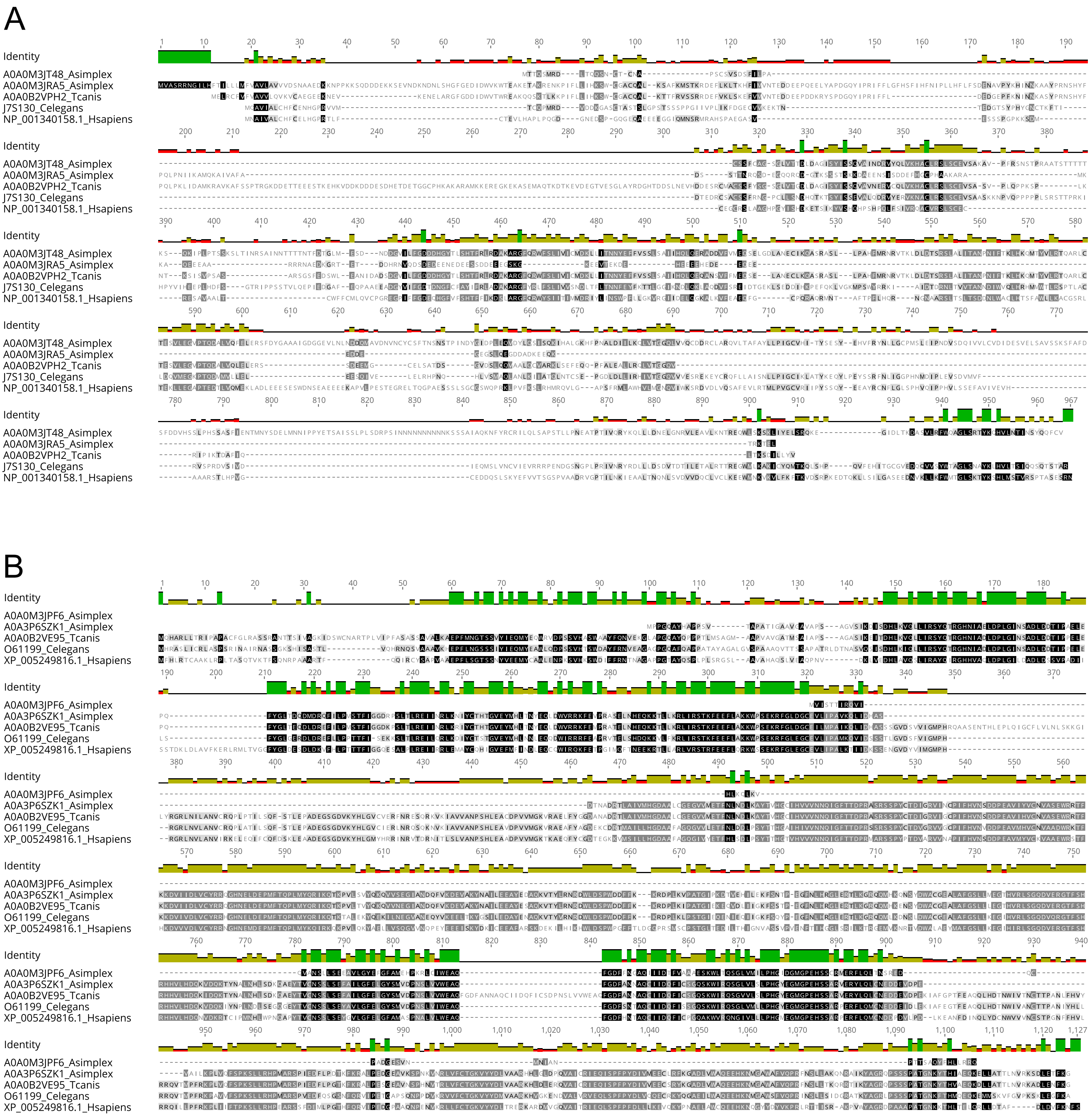

Figure S1: (A , B) Multiple sequence alignment performed to compare the identity of the sequences of the proteins of interest from *A. simplex* s. s., oxoglutarate dehydrogenase (OGDH) and folliculin (FLCN) with the same proteins from *H. sapiens*, *T. canis*, and *C. elegans*.
