# Supplementary material for: Tandem Mass Tagging (TMT) Reveals Tissue-Specific Proteome of L4 Larvae of Anisakis simplex s. s.: Enzymes of Energy and/or Carbohydrate Metabolism as Potential Drug Targets in Anisakiasis
Source: Int J Mol Sci. 2022 Apr 14;23(8):4336. doi: 10.3390/ijms23084336 (PMC9027741; doi:10.3390/ijms23084336)
Supplement: Supplementary file 1 [file ijms-23-04336-s001.zip › ijms-1675296-Supplementary/Figure S2.pdf]

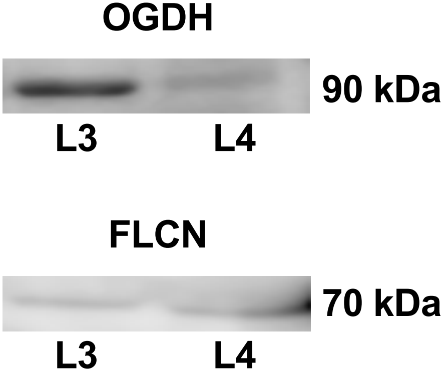

Figure S2: Western blot analysis of the presence of oxoglutarate dehydrogenase (OGDH) and folliculin (FLCN) in *A. simplex* s. s. protein extracts from L3 and L4 stage larvae. The WB analysis using specific primary antibodies for OGDH (diluted 1:1000) and FLCN (diluted 1:1000) was performed as described in Materials and Methods.
