# Supplementary material for: Tandem Mass Tagging (TMT) Reveals Tissue-Specific Proteome of L4 Larvae of Anisakis simplex s. s.: Enzymes of Energy and/or Carbohydrate Metabolism as Potential Drug Targets in Anisakiasis
Source: Int J Mol Sci. 2022 Apr 14;23(8):4336. doi: 10.3390/ijms23084336 (PMC9027741; doi:10.3390/ijms23084336)
Supplement: Supplementary file 1 [file ijms-23-04336-s001.zip › ijms-1675296-Supplementary/Supplementary Materials Captions and Explanations.pdf]

Supplementary Materials Captions and Explanations

File S1:
1. Scheme of sampling and TMT labeling.
2. List of all proteins identified in the study.
3. List of only characterized proteins identified in the study (without uncharacterized proteins).
4. List of DRPs between CUT and REST.
5. List of DRPs between INT and REST.
6. List of DRPs between CUT and INT.

File S2: Results of GO enrichment analysis.
File S3: Results of KEGG pathway enrichment analysis.
File S4: Results of enzyme identification and its classes annotation.
File S5: The characteristics of sequences selected for tertiary structure modeling.
File S6: Results of STRING protein-protein interactions analysis.
File S7: Results of identification of proteins involved in host-parasite interactions.
File S8: Results of identification of potential new allergens.

File S9:
1. List of all proteins identified for L3 and L4 stages of *A. simplex* s. s.
2. List of DRPs between L3 stage and L4 stage.

Figure S1: (A,B) Multiple sequence alignment performed to compare the identity of the sequences of the proteins of interest from *A. simplex* s. s., oxoglutarate dehydrogenase (OGDH) and folliculin (FLCN) with the same proteins from *H. sapiens*, *T. canis*, and *C. elegans.*

Figure S2: Western blot analysis of the presence of oxoglutarate dehydrogenase (OGDH) and folliculin (FLCN) in *A. simplex* s. s. protein extracts from L3 and L4 stage larvae. The WB analysis using specific primary antibodies for OGDH (diluted 1:1000) and FLCN (diluted 1:1000) was performed as described in Materials and Methods.

Figure S3: The volcano plot representation of DRPs between L3 and L4 stages of *A. simplex* s. s.
