# Supplementary figures and images for: Tandem Mass Tagging (TMT) Reveals Tissue-Specific Proteome of L4 Larvae of Anisakis simplex s. s.: Enzymes of Energy and/or Carbohydrate Metabolism as Potential Drug Targets in Anisakiasis
Source: Int J Mol Sci. 2022 Apr 14;23(8):4336. doi: 10.3390/ijms23084336 (PMC9027741; doi:10.3390/ijms23084336)

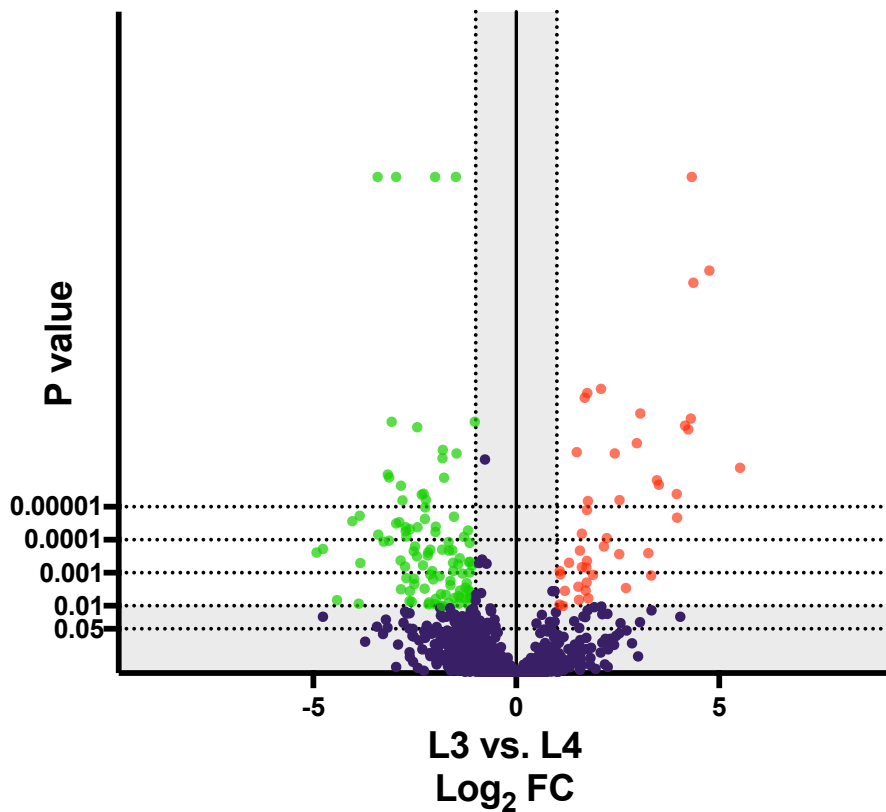

**Figure S3:** The volcano plot representation of DRPs between L3 and L4 stages of *A. simplex s. s.*

Supplement: Supplementary file 1 [file ijms-23-04336-s001.zip › ijms-1675296-Supplementary/Figure S3.pdf]
